# Evaluation of Intratumoral Response Heterogeneity in Metastatic Colorectal Cancer and Its Impact on Patient Overall Survival: Findings from 10,551 Patients in the ARCAD Database

**DOI:** 10.3390/cancers15164117

**Published:** 2023-08-15

**Authors:** Fang-Shu Ou, Daniel H. Ahn, Jesse G. Dixon, Axel Grothey, Yiyue Lou, Pashtoon M. Kasi, Joleen M. Hubbard, Eric Van Cutsem, Leonard B. Saltz, Hans-Joachim Schmoll, Richard M. Goldberg, Alan P. Venook, Paulo Hoff, Jean-Yves Douillard, J. Randolph Hecht, Herbert Hurwitz, Cornelis J. A. Punt, Miriam Koopman, Carsten Bokemeyer, Charles S. Fuchs, Eduardo Diaz-Rubio, Niall C. Tebbutt, Chiara Cremolini, Fairooz F. Kabbinavar, Tanios Bekaii-Saab, Benoist Chibaudel, Takayuki Yoshino, John Zalcberg, Richard A. Adams, Aimery de Gramont, Qian Shi

**Affiliations:** 1 Department of Quantitative Health Sciences, Mayo Clinic, Rochester, MN 55905, USA; 2 Division of Medical Oncology, Mayo Clinic, Phoenix, AZ 85259, USA; 3 West Cancer Center, University of Tennessee, Memphis, TN 38104, USA; 4 Vertex Pharmaceuticals, Boston, MA 02210, USA; 5 Division of Hematology and Oncology, University of Iowa, Iowa City, IA 52242, USA; 6 Department of Oncology, Mayo Clinic, Rochester, MN 55905, USA; 7 Department of Gastroenterology/Digestive Oncology, University Hospitals Gasthuisberg Leuven and KU Leuven, 3000 Leuven, Belgium; 8 Memorial Sloan Kettering Cancer Center, New York, NY 10065, USA; 9 Department of Internal Medicine, Clinic for Internal Medicine IV, Martin-Luther-University Halle/Saale, 06120 Halle, Germany; 10 West Virginia University Cancer Institute, West Virginia University, Morgantown, WV 26506, USA; 11 Department of Medicine, The University of California San Francisco, San Francisco, CA 94143, USA; 12 Department of Clinical Oncology, University of Sao Paulo, Sao Paulo 05508-010, Brazil; 13 Department of Medical Oncology, University of Nantes Medical School, 44035 Nantes, France; 14 Ronald Reagan UCLA Medical Center, Los Angeles, CA 90095, USA; 15 Duke Cancer Institute, Duke University, Durham, NC 27710, USA; 16 Julius Center, University Medical Centre Utrecht, Utrecht University, 3584 CG Utrecht, The Netherlands; 17 Department of Medical Oncology, University Medical Center Utrecht, Utrecht University, 3584 CX Utrecht, The Netherlands; 18 Department of Oncology, Hematology and Bone Marrow Transplantation with Section of Pneumology, University Medical Center Hamburg-Eppendorf, 20251 Hamburg, Germany; 19 Yale Cancer Center, New Haven, CT 06510, USA; 20 Department of Oncology, Hospital Clínico San Carlos, 28040 Madrid, Spain; 21 Sydney Medical School, University of Sydney, Sydney, NSW 2050, Australia; 22 Department of Translational Research and New Technologies in Medicine and Surgery, University of Pisa, 56126 Pisa, Italy; 23 Cardiff Oncology, San Diego, CA 92121, USA; 24 Department of Medical Oncology, Franco-British Institute, 92300 Levallois-Perret, France; 25 Department of Gastrointestinal Oncology, National Cancer Center Hospital East, Chiba 277-8577, Japan; 26 School of Public Health and Preventative Medicine, Monash University, Melbourne, VIC 3004, Australia; 27 Centre for Trials Research, Cardiff University, Cardiff CF14 4YS, UK; 28 Velindre Cancer Center, Velindre NHS Trust, Cardiff CF14 2TL, UK

**Keywords:** tumor measurement-based endpoints, cancer trials

## Abstract

**Simple Summary:**

In colon cancer clinical trials, treatment response is determined from the overall tumor measurement by summing up the individual lesion measurements. However, varied inter-tumor or individual tumor responses are commonly observed in clinical practice. Varied responses are well characterized in clinical trials when measuring one’s response to treatment but its impact on clinical outcomes is unknown. To examine this question, we looked at patients that were enrolled in first-line clinical trials in metastatic colorectal cancer and measured individual lesion changes from their baseline measurements to 12 weeks. Varied responses were very common and occurred in more than 50% of patients. Associations between individual lesion response and patient outcomes were observed where overall survival varied (better or worse) based on the most commonly observed lesion response. A lesion-based criterion demonstrates some of the limitations in the way we currently measure treatment response in clinical trials and could be helpful for treatment decision-making and understanding the prognosis of patients.

**Abstract:**

Metastatic colorectal cancer (mCRC) is a heterogeneous disease that can evoke discordant responses to therapy among different lesions in individual patients. The Response Evaluation Criteria in Solid Tumors (RECIST) criteria do not take into consideration response heterogeneity. We explored and developed lesion-based measurement response criteria to evaluate their prognostic effect on overall survival (OS). Patients and Methods: Patients enrolled in 17 first-line clinical trials, who had mCRC with ≥ 2 lesions at baseline, and a restaging scan by 12 weeks were included. For each patient, lesions were categorized as a progressing lesion (PL: > 20% increase in the longest diameter (LD)), responding lesion (RL: > 30% decrease in LD), or stable lesion (SL: neither PL nor RL) based on the 12-week scan. Lesion-based response criteria were defined for each patient as follows: PL only, SL only, RL only, and varied responses (mixture of RL, SL, and PL). Lesion-based response criteria and OS were correlated using stratified multivariable Cox models. The concordance between OS and classifications was measured using the C statistic. Results: Among 10,551 patients with mCRC from 17 first-line studies, varied responses were noted in 51.6% of patients, among whom, 3.3% had RL/PL at 12 weeks. Among patients with RL/SL, 52% had stable disease (SD) by RECIST 1.1, and they had a longer OS (median OS (mOS) = 19.9 months) than those with SL only (mOS = 16.8 months, HR (95% CI) = 0.81 (0.76, 0.85), *p* < 0.001), although a shorter OS than those with RL only (mOS = 25.8 months, HR (95% CI) = 1.42 (1.32, 1.53), *p* < 0.001). Among patients with SL/PL, 74% had SD by RECIST 1.1, and they had a longer OS (mOS = 9.0 months) than those with PL only (mOS = 8.0 months, HR (95% CI) = 0.75 (0.57, 0.98), *p* = 0.040), yet a shorter OS than those with SL only (mOS = 16.8 months, HR (95% CI) = 1.98 (1.80, 2.18), *p* < 0.001). These associations were consistent across treatment regimen subgroups. The lesion-based response criteria showed slightly higher concordance than RECIST 1.1, although it was not statistically significant. Conclusion: Varied responses at first restaging are common among patients receiving first-line therapy for mCRC. Our lesion-based measurement criteria allowed for better mortality discrimination, which could potentially be informative for treatment decision-making and influence patient outcomes.

## 1. Introduction

The World Health Organization (WHO) response guidelines [1], along with the more recent Response Evaluation Criteria in Solid Tumors (RECIST 1.0) [2], were developed as standardized tools to assess treatment responses in oncological clinical trials. Based on imaging modalities that are readily available and interpretable by investigators, RECIST comprises a standardized set of rules for response measurement using tumor diameter changes to provide a framework for reproducible assessment and analysis. The response assessment tools help to determine whether treatment should be continued or altered for individual patients and to form endpoints for evaluating treatment effects in clinical trials. The radiographic endpoints defined by RECIST, such as objective response rate and complete response, have been utilized in therapeutic development research and are suitable as supportive data for the regulatory approval of novel anticancer treatments by health authorities, such as the US Food and Drug Administration (FDA) [3] and the European Medicines Agency (EMA) [4].

Despite the widespread adoption of RECIST, conventional RECIST classifications were developed to measure responses to conventional chemotherapy. However, developments and changes in the mechanism of action of novel therapies, the use of contemporary imaging modalities, and the adoption of innovative clinical trial designs and study endpoints have necessitated continuous revisions to the tumor assessment criteria. In 2009, the updated RECIST 1.1 [5] included a reduction in the number of lesions to be assessed, as well as new measurement criteria to assess pathologic lymph nodes and elucidate response criteria, and disease progression. The modified RECIST (mRECIST) was developed to assess tumor responses based on viable tumor tissue contrast uptake in the arterial phase of contrast-enhanced imaging in hepatocellular carcinoma [6]. Given the unique tumor response patterns and inability to assess the observed pseudo progression using immunotherapy agents, immune-related RECIST (irRECIST) [7] and, more recently, guidelines for response criteria for trials testing immunotherapeutics (iRECIST) [8] were developed to standardize the response assessment in cancer immunotherapy trials. While changes in tumor size have been associated with treatment response, this does not take into account treatment-associated tumor necrosis. The Choi criteria [9] included tumor size or tumor attenuation, which correlated better than RECIST for gastrointestinal stromal tumors and may be more sensitive for other solid tumors, such as colorectal cancer.

Recent discoveries have led to a more in-depth understanding of the biology of cancers, including insights into tumor heterogeneity, and intercellular differences based on the clonal origin or presence within subpopulations of cancer cells [10]. Metastatic colorectal cancer (mCRC) has been identified to include inter- and intratumoral heterogeneity [11,12,13,14]. In the latter, a “mixed response”, which is an observed discordance in treatment responses among different lesions of the same tumor within an individual patient, is commonly observed in clinical practice and has been acknowledged and described in the literature [15,16]. A heterogeneous intratumoral response has become more evident with the inclusion of targeted therapies, such as biologic agents. Additionally, temporal heterogeneity is acquired over time, as some but not all lesions acquire mechanisms of resistance, especially to targeted therapies, such as anti-EGFR agents; this phenomenon is less pronounced with pure chemotherapy regimens.

Due to the rudimentary nature of tumor assessment, which entails summing the measurements from all target lesions, inherent intratumoral heterogeneity may be concealed; thus, the treatment effect in “mixed responses” may be under-represented. Thus, by acknowledging the importance of intratumoral heterogeneity, the evaluation and quantification of individual tumor lesion responses can potentially have a significant impact on determining how best to define the response to treatment, allow improved decision-making for individual patient therapies, and ultimately, improve patient outcomes. For this analysis, we used the term “varied responses” to refer to the differential responses to treatment within an individual patient, which included three possibilities: (1) some lesions reduced in size (>30%) and others increased in size (>20%), (2) some lesions reduced in size and others remained stable, or (3) some lesions increased in size and others remained stable.

The aims of this study were as follows: (1) to develop a new response criterion incorporating the varied responses of tumors, which is practical in the clinical setting, (2) to quantify the varied response patterns in clinical trial data using this new response criterion, and (3) to evaluate its performance in predicting overall survival (OS). Since this is the first attempt to develop a new criterion, we focused on tumor measurement from the baseline and the first re-staging scan (around 12 weeks).

## 2. Patients and Methods

### 2.1. Population Analysis

The Analysis and Research in Cancers of the Digestive System (ARCAD) is a worldwide collaboration of clinicians, statisticians, and scientists who specialize in gastrointestinal malignancies [17]. The database created by the ARCAD Foundation contains patient-level data from clinical trials that enrolled patients with mCRC from 1997 to 2013. All clinical trials included in the ARCAD database had their study protocols approved by their respective independent ethics committees and the institutional review boards of participating institutions. All patients provided written informed consent to the respective clinical trials that they enrolled in. In the present analysis, patients from first-line trials in the ARCAD database with cycle-by-cycle tumor measurement information and corresponding overall survival data were included. Since this analysis focused on the heterogeneity of early tumor response within individuals, patients with only a single target lesion at baseline and patients who did not have a post-baseline scan prior to 12 weeks were excluded.

### 2.2. Cycle-by-Cycle Tumor Measurements

Among the studies included in this analysis, most of them used RECIST 1.0 for collection and assessment, while the rest utilized the WHO criteria. To harmonize the data collected from different response criteria, several processes were followed:

1. Only data from a maximum of five target lesions were used without considering non-measurable lesions, as we do not have numeric measurements associated with non-measurable lesions. In cases with more than five target lesions, the largest ones were selected to evaluate the response.
2. We only utilized the measurement of the longest diameter per lesion since the current standard response criteria (RECIST 1.1) are based on unidimensional measurements.
3. New lesion information was not used for this analysis because it was not consistently available across trials (it was only available in four trials).
4. The image-based assessment schedule was slightly different across trials; therefore, we considered any assessments that occurred between baseline and 12 weeks from registration. If multiple assessments were available, we chose the assessment that was closest to 12 weeks and included the complete set of tumor measurements.

The 12-week measurements used in this analysis comprise the harmonized data after the above data processing. The RECIST measurements (per RECIST 1.1) used in this analysis were calculated using this harmonized 12-week measurement data without considering new lesion information.

### 2.3. Definition of Lesion-Based Response Criteria at 12 Weeks (LBR12)

To determine the lesion-based tumor response for each patient, we utilized the following two-step process.

Step 1: Classify individual lesions in each patient.

**Figure 1:**
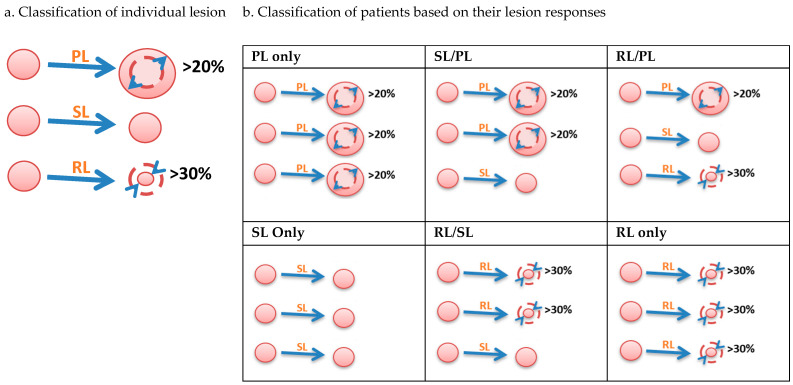
Defining lesion-based response (LBR) criteria. (**a**) Step 1: classify individual lesions within a patient; (**b**) step 2: classify patients into 6 growth patterns. PL: progressing lesion; SL: stable lesion; RL: responding lesion (including complete disappearance).

Based on their measurement at baseline and 12 weeks, we classified lesions into 3 groups: progressing lesion (PL), stable lesion (SL), or responding lesion (RL). PL indicated a 20% increase from baseline, RL indicated a 30% reduction from baseline (including complete disappearance), and SL indicated a less than 20% increase and less than 30% reduction from baseline (Figure 1a).

Step 2: Classify patients into six growth patterns.

It is straightforward to classify patients if every lesion responds to treatment uniformly. We classify this type of patient as PL only, SL only, or RL only. Since multiple lesions within a patient can respond differently to treatment, patients with varied responses to treatment are classified using the best and worst lesion responses. For example, if a patient had three lesions and their responses are classified as RL, SL, and PL, then, the patient is classified as RL/PL (Figure 1b).

Based on this two-step process, we can classify patients into six distinct growth patterns: RL only, RL/SL, SL only, RL/PL, SL/PL, or PL only. In this analysis, we used the term “varied responses” to refer to patients categorized as SL/PL, RL/PL, or RL/SL since these groups represent a heterogeneous response to treatment, among which RL/PL is the most heterogeneous.

### 2.4. Primary Endpoint

The primary endpoint was overall survival (OS), which is defined as the time from the re-staging scan used to define LBR12 until death occurs from any cause.

### 2.5. Statistical Analysis

Baseline clinical characteristics were compared across patients with different growth patterns. Continuous variables were presented as medians with interquartile percentiles, while categorical variables were expressed as counts and percentages. Univariate comparisons were performed using Kruskal–Wallis tests [18] for continuous variables and Pearson Chi-squared tests [19] for categorical variables. The distribution of overall survival was estimated by Kaplan–Meier (KM) [20] curves and the comparison across LBR12 groups was performed using the stratified log-rank test [21]. We used stratified multivariable Cox models [22] to assess the prognostic associations of LBR12 with overall survival, adjusting for other factors (age, sex, and ECOG performance status). Landmark analysis was performed when the date of the re-staging scan, based on which LBR12 was defined, was considered as the landmark time. The analysis was repeated within each patient subgroup, defined by the treatment regimen, chemo alone, with a vascular endothelial growth factor inhibitor (VEGFi), and epidermal growth factor receptor inhibitors (EGFRi). To understand the effects of responding and progressing lesions among patients who were identified as RL/PL, we further categorized them based on whether the responding or progressing lesions were the most prevalent, i.e., patients were separated based on more responding lesions, equal numbers of responding and progressing lesions, or more progressing lesions. To understand the additional prognostic effect of LBR12 among patients with the same RECIST 1.1 classification, we investigated the association between LBR12 and OS in patient subgroups defined by RECIST 1.1. The goodness of fit of the survival models was measured by concordance statistics. [23] A 2-sided *p*-value of < 0.05 was considered statistically significant for all tests. No adjustments were made for multiple comparisons since all analyses were considered exploratory. All analyses were performed using SAS software version 9.4 (SAS Institute, Cary, NC, USA).

## 3. Results

**Figure 2:**
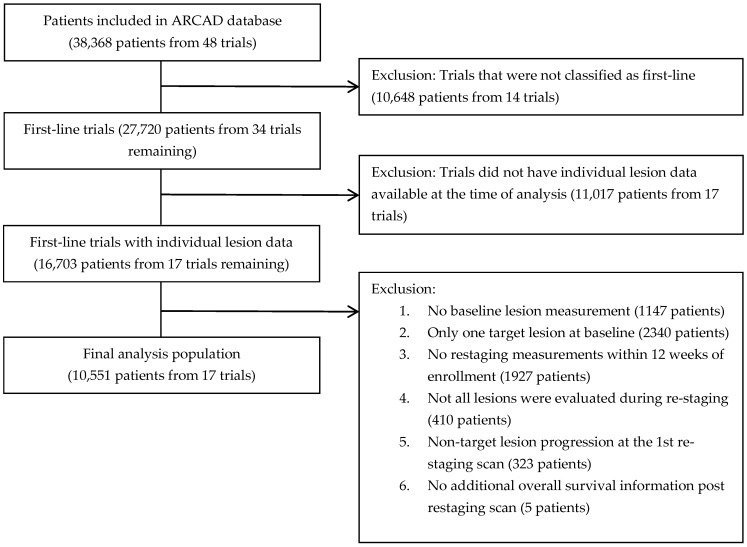
Analysis population.

Among all patients included in the ARCAD database, 10,648 patients from 14 trials were excluded because they were not enrolled in 1st-line trials, 11,017 patients from 17 trials were excluded because they did not have individual lesion data available at the time of analysis, and an additional 6152 patients from the remaining 17 trials (Table S1) were excluded for the following reasons: no baseline measurements, only 1 targeted lesion recorded at baseline, no re-staging measurement within 12 weeks of enrollment, not all lesions were evaluated during re-staging, progression due to non-target lesion at the 1st re-staging, or no additional survival information available post-re-staging. Finally, 10,551 patients enrolled in 17 mCRC 1st-line trials were included in this analysis (Figure 2).

### 3.1. High Proportion of Patients Had Heterogeneous Tumor Responses

Among the 10,551 patients included in this analysis, 69 (0.7%) were categorized as PL only, 665 (6.3%) were categorized as SL/PL, 349 (3.3%) were categorized as RL/PL, 3276 (31.0%) were categorized as SL only, 4429 (42.0%) were categorized as RL/SL and 1763 (16.7%) were categorized as RL only, according to our lesion-based response criteria. Overall, 51.6% of patients (N = 5443) had varied responses in terms of lesion size changes at 12 weeks of treatment (i.e., they were categorized as SL/PL, RL/PL, or RL/SL), while 3.3% of patients (N = 349) had the most extreme varied responses (i.e., some lesions responded to treatment, yet others did not, RL/PL).

Baseline characteristics according to the lesion-based response category are listed in Table 1. There were no clinically relevant differences in age, gender, liver metastasis, number of lesions at baseline, or median diameter of baseline lesions among patients with different lesion growth patterns. The demographic and clinical characteristics of patients included in this analysis versus those who were not were compared descriptively (Table S2). Since this analysis included patients with at least two lesions at baseline, they had a higher disease burden (i.e., they were more likely to have liver or lung involvement and more metastatic sites) than those who were not; otherwise, no clinically relevant differences in age, gender, and performance status were noted.

Patients who received a VEGF inhibitor (VEGFi) or EGFR inhibitor (EGFRi) had higher rates of varied responses (52.7% and 52.4%) compared to those who had chemotherapy alone (49.8%). The RL/PL rates were similar among patients treated with chemotherapy alone, VEGFi, and EGFRi (3.7%, 3.0%, and 3.1%, respectively). Patients who received EGFRi had the highest rate of RL only (23.5%) compared to patients who received VEGFi or chemotherapy alone. Patients who received VEGFi had the highest rate of RL/SL (45.1%) compared to patients who received EGFRi (44.0%) or chemotherapy alone (37.6%). Patients who had lung involvement and more metastatic sites also had high rates of varied responses (54.3% and 55.0%, respectively).

### 3.2. Overall Survival Increased across Patients Who Had More RLs and Fewer PLs

**Figure 3:**
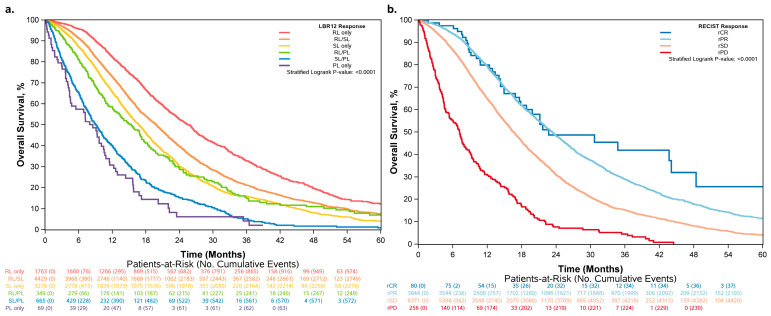
Overall survival by (**a**) LBR12 and (**b**) RECIST 1.1.

**Figure 4:**
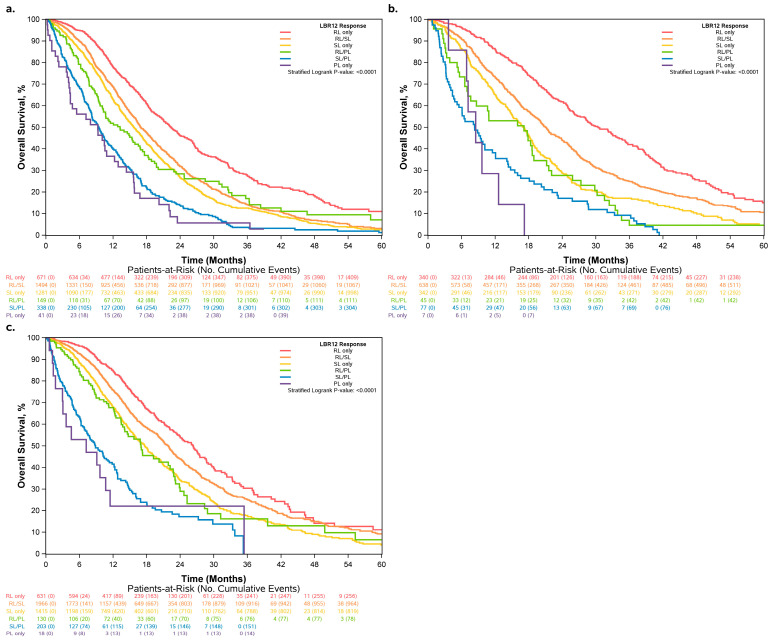
Overall survival by LBR12 within regimen subgroups: (**a**) patients who received chemotherapy only, (**b**) patients who received EGFRi, and (**c**) patients who received VEGFi.

The KM curves for overall survival by LBR12 category are shown in Figure 3a. The median OS increased for patients with more RLs and fewer PLs, while the median OS was the shortest (8.0 months) among PL-only patients and the longest (25.8 months) among RL-only patients (Table 2). This pattern of increasing OS remained the same for patients who received chemotherapy alone and VEGF inhibitors (Figure 4). For patients who received the EGFR inhibitor, the median OS for those with PL only was slightly longer (8.5 months) than those with SL/PL (8.3 months); however, this inconsistency diminished after multivariable adjustment (Table 2). In the overall population, comparisons across two adjacent LBR12 levels showed clinically meaningful (hazard ratio > 1.2) and statistically significant differences for all comparisons, except RL/PL vs. SL (Table S3). The magnitude of HR remained similar among patient subgroups as defined by the treatment regimen, although the *p*-values tended to be less significant in these subgroups with smaller sample sizes.

**Figure 5:**
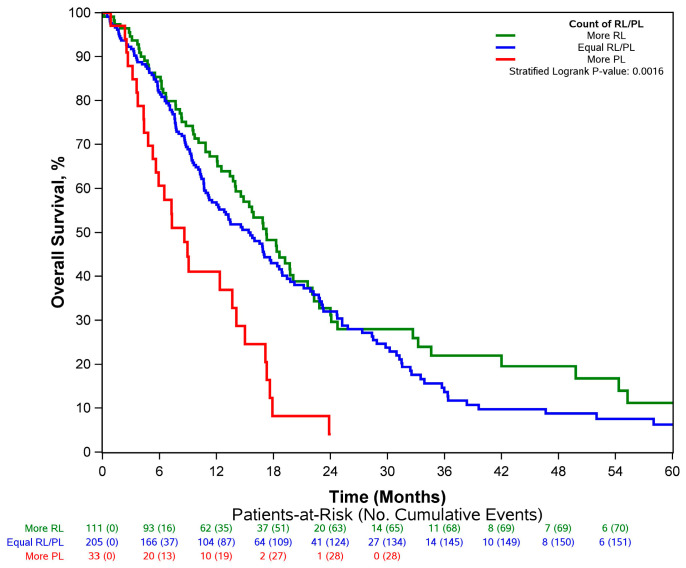
Overall survival by most prevalent tumor response among patients categorized as RL/PL.

Among patients categorized as RL/PL (N = 349), 111 patients had more responding lesions than progressing lesions, 33 patients had equal numbers of responding and progressing lesions, and 205 patients had more progressing than responding lesions. Even though all of these patients belonged to the same RL/PL group per our definition, the number of progressing lesions observed was negatively associated with OS (Figure 5). Patients with more progressing lesions had worse outcomes (median OS = 8.6 months) even within the same RL/PL group (median OS = 15.4 months for patients with equal numbers of progressing and responding lesions; median OS = 17.2 months for patients with more responding than progressing lesions) (Table 3).

### 3.3. Differences in Classification by RECIST 1.1 vs. LBR12

The KM curves for overall survival by RECIST 1.1 and LBR12 are shown side-by-side in Figure 3 to provide a visual comparison of the two classifications. The median OS for complete response (CR), partial response (PR), stable disease (SD), and progressive disease (PD) by RECIST 1.1 is 22.7, 23.4, 16.6, and 7.2 months, respectively.

Patients classified as RL only, SL only, or PL only also had complete/partial response, stable disease, and progressive disease at 12 weeks by RECIST 1.1, respectively (Figure S1). Among patients classified as RL/SL, 52.5% had SD by RECIST 1.1. Among patients classified as SL/PL, 73.8% had SD by RECIST 1.1. The majority of patients with the most heterogeneous responses (RL/PL) were determined to have SD (79.9%) by RECIST 1.1, while others had PR (16.3%) or PD (3.7%).

**Figure 6:**
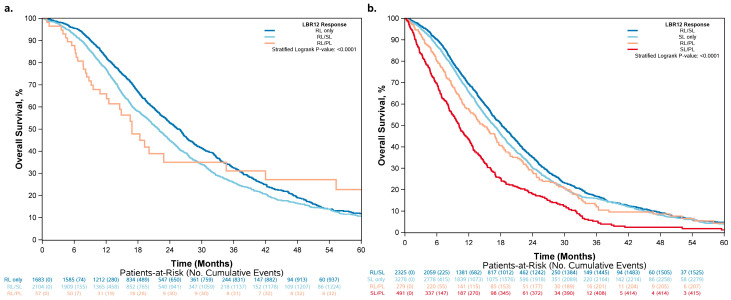
Overall survival by LBR12: (**a**) patients with partial response by RECIST 1.1; (**b**) patients with stable disease by RECIST 1.1.

LBR12 provides additional risk stratification even when patients have the same response according to RECIST 1.1. Among patients determined to have a partial response by RECIST 1.1 (N = 3844), patients classified as RL/PL had a worse outcome (median OS = 16.9 months) compared to those classified as RL/SL (median OS = 21.9 months) and RL only (median OS = 25.7 months), with the latter having the best outcome (Figure 6 and Table 4). Similarly, among patients determined to have a stable disease by RECIST 1.1 (N = 6371), those classified as SL/PL had the worst outcome (median OS = 10.2 months) and those classified as RL/SL had the best outcome (median OS = 18.2 months) (Figure 6 and Table 4).

### 3.4. LBR12 Produced a Higher Concordance Rate for Overall Survival Than RECIST at 12 Weeks

The concordance rates of LBR12 and RECIST 1.1 for overall survival are shown in Table 5. In the overall analysis population, and in each regimen subgroup, LBR12 produced a slightly higher concordance rate for overall survival than RECIST 1.1, even though the difference was not statistically significant (i.e., the 95% CI of the concordance by LBR12 overlaps the concordance by RECIST, and vice versa).

### 3.5. Sensitivity Analysis with Varying Definitions of LBR12

Six sensitivity analyses were conducted using different cutoff points to investigate whether the chosen cutoffs influenced the results. The six combinations were as follows: (1) ≥20% increase as PL, <25% decrease as RL; (2) ≥20% increase as PL, <20% decrease as RL; (3) ≥15% increase as PL, <30% decrease as RL; (4) ≥15% increase as PL, <25% decrease as RL; (5) ≥10% increase as PL, <30% decrease as RL; and (6) ≥10% increase as PL, <20% decrease as RL. The different cutoffs did not have a major impact on the classification and corresponding association with OS.

## 4. Discussion

Colorectal cancer is a heterogeneous disease with vast inter- and intratumoral differences. Molecular and biological variations observed among tumors and in subclones within tumors can result in discordant tumor responses to treatment between lesions in the same cancer within an individual patient. With the emergence of novel therapies with unique mechanisms of action, including biologically and molecularly targeted agents [24,25,26,27], heterogeneous tumor responses are more evident. Tumor heterogeneity can modulate disease progression and treatment resistance through biological interactions between subclones [28]. While these findings have been observed in clinical practice and described in the literature, no systematic studies have been conducted to describe the biological impact on patient outcomes. These factors help support the rationale for performing this study to evaluate and understand the impact of tumor lesion responses within individual patients on clinical outcomes.

The findings of our study demonstrate that lesion-based responses highlight the heterogeneity observed in tumor responses within individual patients, with the majority of patients (52%) having heterogeneous tumor responses to treatment in terms of lesion size changes. Significant differences in overall survival were noted between patients with tumors that demonstrated a “varied response” and those with homogenous changes in tumor lesion measurements, even within patients with the same response category by RECIST 1.1. Individual tumor responses further divide the RECIST response into different categories, demonstrating that patients in the same RECIST response category can have very different outcomes depending on their lesion-level response. Among patients whom we defined as RL/PL, whether the majority of target lesions were responding or progressing lesions, these were also associated with different OS. These results suggest that an individual tumor response and lesion heterogeneity are prognostic for patient survival. Patients with stable disease by RECIST 1.1 but considered to be in the SL/PL group may benefit from a change in therapy prior to disease progression. The OS outcomes of this group (median OS = 10.2 months) were more similar to patients with progressive disease by RECIST 1.1 (median OS = 7.2 months; Figure 3b) than other patients with stable disease by RECIST 1.1 (median OS = 14.7, 16.7, and 18.2 months; Figure 6b). However, prospective randomized trials are needed to validate these observations.

The high proportion of heterogeneous intratumoral lesion responses observed in patients who received biological (anti-VEGF) and targeted (anti-EGFR) agents reinforced the potential significance of intratumoral heterogeneity and are potentially the result of secondary acquired resistance through clonal evolution and the emergence of treatment-resistant mutant clones with unique alterations against these agents [29,30,31]. The observed survival differences among patients who had heterogeneous responses, in terms of lesion size, suggest a potential benefit to continuing treatment beyond progression per RECIST, which has been observed when using targeted agents in clinical trials [32,33].

For the lesion-based response criteria, we used an increase of >20% and a decrease of <30% (including the complete disappearance of a lesion) to define lesion-based progression and partial response, respectively. The tumor measurement cutoffs for the lesion-based response criteria were taken from RECIST 1.1, the standard utilized in clinical trials. A separate category for lesions that were defined as showing a complete response was not delineated in the lesion-based response criteria due to the low incidence and lack of impact on clinical treatment. There were no changes in treatment for patients in the included trials who experienced either partial or complete response; both continued with their current therapy.

Since this was the first attempt to develop new lesion-based response criteria, we only focused on the 12-week tumor measurement, which was typically based on the 1st or 2nd re-staging scan in this population. It is conceivable that if all the data points from the re-staging scan measurements were used, more complete responses would potentially be included, which would better describe the acquired mechanisms of resistance and “temporal” heterogeneity. The same logic behind the creation of the current lesion-based response criteria would also still apply.

In the ARCAD database, there are currently no data from clinical trials utilizing immunotherapy; therefore, we could not evaluate the performance of the proposed criteria for patients who received immunotherapy. Given that mixed responses have been observed in patients with lung cancer receiving immunotherapy [34,35,36], we conjectured that the new lesion-based criteria would be able to identify patients with mixed responses beyond RECIST 1.1. There will need to be additional analyses to evaluate whether the association with OS observed in this analysis can be generalized to other treatment options, including immunotherapy, and disease settings.

Our study has several limitations. All patients included in this analysis were deemed eligible and appropriate for participation in clinical trials, therefore, they may not be representative of a real-world population. However, the analysis was conducted using data from international multicenter clinical trials, meaning it is likely to be more representative and generalizable compared to studies at single institutions or in the Eastern or Western world. Given that each trial collected different variables, in order to harmonize the data across multiple trials, we could adjust for only a limited set of variables as potential confounders. The trials included in this analysis utilized different response criteria (WHO and RECIST 1.0); therefore, to use the data in a consistent fashion and provide insights on using the current response criteria, we recalculated the response criteria using RECIST 1.1. Thus, we inherently adopted the limitations of RECIST 1.1, including the ability to only assess a maximum of five target lesion measurements and a maximum of two lesions per organ. Since not all of the trials provided new lesion information, we could not use the new lesion information to harmonize the measurement data, which may have resulted in some patients with new lesion progressions being classified into a response group other than PD. Lastly, the lesion measurements were reported by different radiologists without a centralized review, thus, the variability in radiology measurements and reporting could potentially have introduced variance based on individualized assessments.

## 5. Conclusions

In conclusion, based on heterogeneous inter- and intratumoral lesion responses, our findings demonstrate that assessment using lesion-based response criteria was associated with OS, which was consistent across different treatment regimens. Future studies taking into consideration a central review of lesion measurements to validate these findings are warranted and may represent a novel way to assess targeted therapies, including immunotherapy [37,38]. This is increasingly more relevant since more patients are being diagnosed with early onset colorectal cancer in their 20s, 30s, and 40s, and thus, are exposed to more treatments over time, leading to greater tumor heterogeneity.

## Figures and Tables

**Table 1:** Patient characteristics by LBR12.

	RL Only (N = 1763)	RL/SL (N = 4429)	SL Only (N = 3276)	RL/PL (N = 349)	SL/PL (N = 665)	PL Only (N = 69)	Total (N = 10,551)	*p*-Value
Age at Enrollment								<0.0001 ^1^
Missing	1	0	1	0	0	0	2	
Mean (STD)	59 (11.3)	60 (10.9)	60 (11.1)	59 (10.9)	59 (11.6)	58 (11.2)	60 (11.1)	
Median (IQR)	60 (52, 67)	60 (53, 68)	61 (54, 68)	60 (51, 68)	59 (51, 68)	59 (53, 66)	60 (53, 68)	
Range	20, 84	18, 89	18, 88	26, 83	19, 83	24, 79	18, 89	
Gender, n (%)								0.0064 ^2^
Female	712 (40.4%)	1744 (39.4%)	1288 (39.3%)	162 (46.4%)	299 (45.0%)	22 (31.9%)	4227 (40.1%)	
Male	1050 (59.6%)	2685 (60.6%)	1988 (60.7%)	187 (53.6%)	366 (55.0%)	47 (68.1%)	6323 (59.9%)	
Missing	1	0	0	0	0	0	1	
Performance Status, n (%)								< 0.0001 ^2^
0	1089 (62.3%)	2480 (56.2%)	1764 (54.4%)	165 (47.4%)	355 (54.1%)	35 (51.5%)	5888 (56.2%)	
1	642 (36.7%)	1871 (42.4%)	1420 (43.8%)	172 (49.4%)	283 (43.1%)	30 (44.1%)	4418 (42.2%)	
2	17 (1.0%)	60 (1.4%)	60 (1.8%)	11 (3.2%)	18 (2.7%)	3 (4.4%)	169 (1.6%)	
Missing	15	18	32	1	9	1	76	
Treatment Regimen, n (%)								< 0.0001 ^2^
Chemotherapy alone	671 (38.1%)	1494 (33.7%)	1281 (39.1%)	149 (42.7%)	338 (50.8%)	41 (59.4%)	3974 (37.7%)	
VEGFi	631 (35.8%)	1966 (44.4%)	1415 (43.2%)	130 (37.2%)	203 (30.5%)	18 (26.1%)	4363 (41.4%)	
EGFRi	340 (19.3%)	638 (14.4%)	342 (10.4%)	45 (12.9%)	77 (11.6%)	7 (10.1%)	1449 (13.7%)	
VEGFi and EGFRi	121 (6.9%)	331 (7.5%)	238 (7.3%)	25 (7.2%)	47 (7.1%)	3 (4.3%)	765 (7.3%)	
Liver Affected, n (%)								0.0038 ^2^
No	220 (17.0%)	567 (16.9%)	520 (20.6%)	47 (17.0%)	81 (15.6%)	9 (16.7%)	1444 (18.0%)	
Yes	1071 (83.0%)	2780 (83.1%)	2000 (79.4%)	229 (83.0%)	438 (84.4%)	45 (83.3%)	6563 (82.0%)	
Missing	472	1082	756	73	146	15	2544	
Lung Affected, n (%)								<0.0001 ^2^
No	909 (70.6%)	1964 (58.8%)	1508 (60.2%)	170 (62.3%)	344 (67.1%)	40 (76.9%)	4935 (61.9%)	
Yes	379 (29.4%)	1374 (41.2%)	997 (39.8%)	103 (37.7%)	169 (32.9%)	12 (23.1%)	3034 (38.1%)	
Missing	475	1091	771	76	152	17	2582	
N of Metastatic Sites, n (%)								<0.0001 ^2^
0	3 (0.2%)	18 (0.5%)	8 (0.3%)	1 (0.4%)	0 (0.0%)	1 (1.9%)	31 (0.4%)	
1	687 (53.1%)	1288 (38.5%)	1047 (41.6%)	91 (33.0%)	184 (35.5%)	27 (50.9%)	3324 (41.5%)	
2+	603 (46.6%)	2041 (61.0%)	1463 (58.1%)	184 (66.7%)	334 (64.5%)	25 (47.2%)	4650 (58.1%)	
Missing	470	1082	758	73	147	16	2546	
Number of Lesions at Baseline								<0.0001 ^1^
Missing	0	0	0	0	0	0	0	
Mean (STD)	3.3 (1.2)	3.8 (1.2)	3.2 (1.2)	4.1 (1.0)	3.8 (1.2)	2.7 (0.9)	3.5 (1.2)	
Median (IQR)	3.0 (2.0, 4.0)	4.0 (3.0, 5.0)	3.0 (2.0, 4.0)	4.0 (3.0, 5.0)	4.0 (3.0, 5.0)	2.0 (2.0, 3.0)	3.0 (2.0, 5.0)	
Range	2.0, 5.0	2.0, 5.0	2.0, 5.0	2.0, 5.0	2.0, 5.0	2.0, 5.0	2.0, 5.0	
Sum of Baseline Lesion Diameters (cm)								<0.0001 ^1^
Missing	0	0	0	0	0	0	0	
Mean (STD)	11.4 (7.5)	14.5 (8.4)	13.3 (8.7)	14.4 (7.8)	14.0 (8.1)	8.6 (5.6)	13.6 (8.4)	
Median (IQR)	9.5 (5.9, 14.9)	12.7 (8.2, 19.0)	11.1 (6.9, 17.7)	12.7 (8.5, 18.4)	12.7 (7.9, 18.2)	7.7 (5.0, 10.2)	11.7 (7.3, 17.9)	
Range	2.0, 71.5	0.8, 72.3	1.7, 62.5	2.6, 53.2	1.8, 58.6	2.1, 28.0	0.8, 72.3	
Median of Baseline Lesion Diameters (cm)								<0.0001 ^1^
Missing	0	0	0	0	0	0	0	
Mean (STD)	3.4 (2.0)	3.6 (2.0)	3.9 (2.3)	3.1 (1.6)	3.5 (1.9)	3.2 (1.9)	3.6 (2.1)	
Median (IQR)	2.9 (2.0, 4.2)	3.0 (2.1, 4.5)	3.4 (2.2, 5.0)	2.8 (2.0, 3.9)	3.0 (2.2, 4.3)	2.8 (1.8, 4.1)	3.0 (2.1, 4.5)	
Range	0.6, 15.3	0.4, 18.6	0.4, 20.0	0.8, 10.8	0.9, 12.9	1.1, 10.5	0.4, 20.0	

^1^ Kruskal–Wallis *p*-value; ^2^ Chi-squared *p*-value. STD: standard deviation; IRQ: interquartile range; LBR12: lesion-based response criteria at 12 weeks; RL: responding lesion; SL: stable lesion; PL: progressing lesion; EGFRi: epidermal growth factor receptor inhibitor; VEGFi: vascular endothelial growth factor inhibitor.

**Table 2:** Median overall survival and hazard ratio by LBR12 and treatment regimen.

Treatment Regimen	LBR12 Response	Median (95% CI)	Hazard Ratio (95% CI)	*p*-Value
Overall		18.4 (18.1–18.9)		<0.001 ^1^
	RL	25.8 (24.1–26.7)	0.57 (0.53–0.62)	<0.001 ^2^
	RL/SL	19.9 (19.2–20.5)	0.81 (0.76–0.85)	<0.001 ^2^
	SL	16.8 (16.1–17.4)	Reference	
	RL/PL	15.4 (13.2–17.1)	1.10 (0.96–1.25)	0.164 ^2^
	SL/PL	9.0 (8.2–10.1)	1.98 (1.80–2.18)	<0.001 ^2^
	PL	8.0 (4.5–10.7)	2.65 (2.04–3.43)	<0.001 ^2^
Chemotherapy alone		16.5 (16.1–17.0)		<0.001 ^1^
	RL	22.4 (20.9–24.8)	0.60 (0.53–0.67)	<0.001 ^2^
	RL/SL	17.0 (16.3–18.2)	0.87 (0.79–0.95)	0.002^2^
	SL	15.7 (14.9–16.8)	Reference	
	RL/PL	12.8 (10.3–16.8)	1.07 (0.88–1.31)	0.492 ^2^
	SL/PL	9.3 (8.2–10.4)	1.80 (1.57–2.05)	<0.001 ^2^
	PL	8.6 (4.4–14.4)	2.18 (1.56–3.04)	<0.001 ^2^
EGFRi		20.3 (19.4–21.6)		<0.001 ^1^
	RL	30.5 (26.7–34.8)	0.50 (0.42–0.60)	<0.001 ^2^
	RL/SL	21.1 (19.7–22.9)	0.76 (0.65–0.87)	<0.001 ^2^
	SL	17.2 (15.1–18.4)	Reference	
	RL/PL	17.1 (7.6–21.2)	1.28 (0.92–1.77)	0.143 ^2^
	SL/PL	8.3 (5.5–12.1)	2.18 (1.68–2.82)	<0.001 ^2^
	PL	8.5 (6.9-NE)	3.17 (1.48–6.76)	0.003 ^2^
VEGFi		20.2 (19.4–20.9)		<0.001 ^1^
	RL	26.3 (23.8–28.6)	0.57 (0.49–0.66)	<0.001 ^2^
	RL/SL	21.9 (20.9–22.6)	0.77 (0.70–0.85)	<0.001 ^2^
	SL	17.7 (16.5–18.7)	Reference	
	RL/PL	17.0 (14.0–22.8)	1.09 (0.86–1.37)	0.466 ^2^
	SL/PL	8.9 (7.3–11.3)	2.27 (1.91–2.71)	<0.001 ^2^
	PL	7.2 (3.0-NE)	3.73 (2.18–6.37)	<0.001 ^2^

^1^ Stratified type 3 likelihood ratio *p*-value to test differences across groups; ^2^ Wald *p*-value. LBR12: lesion-based response criteria at 12 weeks; RL: responding lesion; SL: stable lesion; PL: progressing lesion; EGFRi: epidermal growth factor receptor inhibitor; VEGFi: vascular endothelial growth factor inhibitor. Variables adjusted in models: age, gender, and ECOG performance status.

**Table 3:** Median overall survival and hazard ratio by most prevalent tumor response among patients categorized as RL/PL.

	Median (95% CI) ^1^	Adjusted Hazard Ratio (95% CI) ^2^	Adjusted *p*-Value
Most prevalent tumor response			0.0148 ^3^
More RL	17.2 (14.6–21.6)	0.93 (0.67–1.28)	
Equal RL/PL	15.4 (12.0–18.6)	Reference	
More PL	8.6 (5.6–15.0)	2.04 (1.25–3.33)	

^1^ Kaplan–Meier method; ^2^ Cox model; ^3^ likelihood ratio test. RL: responding lesion; PL: progressing lesion. Adjusted for age, gender, and ECOG performance status.

**Table 4:** Median overall survival and hazard ratio by LBR12 among patients with partial response or stable disease by RECIST 1.1.

	Median (95% CI) ^1^	Adjusted Hazard Ratio (95% CI) ^2^	Adjusted *p*-Value
Patients with partial response by RECIST 1.1			<0.0001 ^3^
RL	25.7 (24.1–26.7)	Reference	
RL/SL	21.9 (20.6–22.9)	1.26 (1.16–1.38)	
RL/PL	16.9 (12.5–42.0)	1.47 (1.04–2.08)	
Patients with stable disease by RECIST 1.1			<0.0001 ^3^
RL/SL	18.2 (17.5–19.1)	Reference	
SL	16.7 (16.1–17.4)	1.09 (1.02–1.17)	
RL/PL	14.7 (12.8–17.2)	1.26 (1.08–1.45)	
SL/PL	10.2 (9.2–11.3)	1.93 (1.73–2.16)	

^1^ Kaplan–Meier method; ^2^ Cox model; ^3^ likelihood ratio test. RL: responding lesion; PL: progressing lesion. Adjusted for age, gender, and ECOG performance status.

**Table 5:** Concordance from stratified Cox models * for the overall population and patient regimen subgroups.

	Concordance (95% CI)	
	LBR12 ^$^	RECIST 1.1 ^$^
Overall	0.626 (0.583–0.669)	0.617 (0.574–0.660)
Chemotherapy alone	0.618 (0.573–0.664)	0.603 (0.557–0.648)
EGFRi	0.645 (0.611–0.678)	0.641 (0.608–0.674)
VEGFi	0.622 (0.576–0.667)	0.612 (0.567–0.658)

LBR12: lesion-based response criteria at 12 weeks; EGFRi: epidermal growth factor receptor inhibitor; VEGFi: vascular endothelial growth factor inhibitor. * Stratified by treatment arm; adjusted for age, gender, and ECOG performance status. ^$^ LBR12 and RECIST 1.1 were defined based on lesion changes from enrollment to 12 weeks.

## Data Availability

Restrictions apply to the availability of the data. The sharing of individual patient data from each participating trial is subject to the policies and procedures of the institutions and groups that conducted the original studies. Data were obtained from the ARCAD Foundation (https://www.fondationarcad.org/en/, accessed on 11 August 2023).

## Supplementary Materials

The following supporting information can be downloaded at: https://www.mdpi.com/article/10.3390/cancers15164117/s1. Figure S1. Patient classifications by LBR12 vs. RECIST 1.1. Table S1. Information of clinical trials included in this study. Table S2. Patient characteristics by whether patients were included in the analysis population. Table S3. Pair-wise comparison across adjacent LBR12 level.
