# Supplementary material for: Evaluation of Intratumoral Response Heterogeneity in Metastatic Colorectal Cancer and Its Impact on Patient Overall Survival: Findings from 10,551 Patients in the ARCAD Database
Source: Cancers (Basel). 2023 Aug 15;15(16):4117. doi: 10.3390/cancers15164117 (PMC10452983; doi:10.3390/cancers15164117)
Supplement: Supplementary file 1 [file cancers-15-04117-s001.zip › cancers-2483142-supplementary.pdf]

Table S1. Information of clinical trials included in this study

Study	CT.gov Number	Original Manuscript Reference	Treatment Arm Included in Analyses	N Patients Included
BICC-C	NCT00101686	Fuchs, 2007	CAPIRI	86
			FOLFIRI	95
			FOLFIRI + Bev	43
			mIFL	95
			mIFL + Bev	40
AVF2107g	NCT00109070	Hurwitz, 2004	5FULV + Bev	85
			IFL + Bev	328
			IFL + Placebo	313
N016966	NCT00069095	Saltz, 2008	CAPOX	219
			CAPOX + Bev	244
			CAPOX + Placebo	250
			FOLFOX4	210
			FOLFOX4 + Bev	256
			FOLFOX4 + Placebo	259
AVF2192g	NCT00109226	Kabbinavar, 2005	5FULV + Bev	76
			5FULV + Placebo	57
N9741	NCT00003594	Goldberg, 2004	5FU	15
		Goldberg, 2009	5FU + Oxali	24
			FOLFOX	178
			IFL	111
			IROX	174
			mIFL	4
			mIFL (Daily Bolus)	17
			rIFL	63
PACCE (C249)	NCT00115765	Hecht, 2009	FOLFIRI + Bev	52
			FOLFIRI + Bev + Pan	52
			FOLFOX + Bev	183
			FOLFOX + Bev + Pan	185
PRIME (C203)	NCT00364013	Douillard, 2010	FOLFOX4	409
			FOLFOX4 + Pan	399
CAIRO1	NCT00312000	Koopman, 2007	CAPIRI -> CAPOX	199
			Cape -> Iri -> CAPOX	203
CAIRO2	NCT00208546	Tol, 2009	CAPOX + Bev	212
			CAPOX + Bev + Cet	223
CRYSTAL	NCT00154102	Van Cutsem, 2009	FOLFIRI	451
		Van Cutsem, 2011	FOLFIRI + Cet	425
Macro	NCT00335595	Díaz-Rubio, 2012	CAPOX + Bev-> Bev	150
			CAPOX + Bev -> CAPOX + Bev	158
AGITG (MAX)	NCT00294359	Tebbutt, 2010	Cape	112
			Cape + Bev	121
			Cape + Bev + Mitomycin	111
GONO	NCT01219920	Falcone, 2007	FOLFIRI	16
			FOLFOXIRI	26
HORIZON II	NCT00399035	Hoff, 2012	FOLFOX/CAPOX + Cediranib 20 mg	408
			FOLFOX/CAPOX + Cediranib 30 mg	153
			FOLFOX/CAPOX + Placebo	284
HORIZON III	NCT00384176	Schmoll, 2012	mFOLFOX6 + Bev	540
			mFOLFOX6 + Cediranib 20mg	511
			mFOLFOX6 + Cediranib 30mg	147
OPUS	NCT00125034	Bokemeyer, 2009	FOLFOX	104
		Bokemeyer, 2011	FOLFOX + Cet	116

Study	CT.gov Number	Original Manuscript Reference	Treatment Arm Included in Analyses	N Patients Included
CALGB-80405	NCT00265850	Venook, 2017	Chemo + Bev	545
			Chemo + Bev + Cet	305
			Chemo + Cet	509

Table S2. Patient characteristics by whether patients were included in the analysis population

	Analysis Population No (N=6152)	Yes (N=10551)	Total (N=16703)	P-value
**Age at enrollment**				<.0001[1]
Mean (SD)	60.9 (11.23)	59.6 (11.09)	60.1 (11.16)	
Median (IQR)	62.0 (54.0, 69.0)	60.0 (53.0, 68.0)	61.0 (53.0, 68.0)	
Range	18.0, 90.0	18.0, 89.0	18.0, 90.0	
**Gender**, n (%)				0.2864[2]
Female	2510 (40.9%)	4227 (40.1%)	6737 (40.4%)	
Male	3626 (59.1%)	6323 (59.9%)	9949 (59.6%)	
Missing	16	1	17	
**Performance Score**, n (%)				0.0013[2]
0	3386 (56.3%)	5888 (56.2%)	9274 (56.2%)	
1	2482 (41.3%)	4418 (42.2%)	6900 (41.8%)	
2	146 (2.4%)	169 (1.6%)	315 (1.9%)	
4	1 (0.0%)	0 (0.0%)	1 (0.0%)	
Missing	137	76	213	
**Regimens**, n (%)				<.0001[2]
Chemo Alone	2673 (43.4%)	3974 (37.7%)	6647 (39.8%)	
VEGFi	1983 (32.2%)	4363 (41.4%)	6346 (38.0%)	
EGFRi	824 (13.4%)	1449 (13.7%)	2273 (13.6%)	
VEGFi & EGFRi	672 (10.9%)	765 (7.3%)	1437 (8.6%)	
**Liver Affected**, n (%)				<.0001[2]
No	1303 (28.6%)	1444 (18.0%)	2747 (21.9%)	
Yes	3247 (71.4%)	6563 (82.0%)	9810 (78.1%)	
Missing	1602	2544	4146	
**Lung Affected**, n (%)				<.0001[2]
No	3079 (68.5%)	4935 (61.9%)	8014 (64.3%)	
Yes	1418 (31.5%)	3034 (38.1%)	4452 (35.7%)	
Missing	1655	2582	4237	
**N of Metastatic Sites**, n (%)				<.0001[2]
0	63 (1.4%)	31 (0.4%)	94 (0.7%)	
1	2298 (50.5%)	3324 (41.5%)	5622 (44.8%)	
2+	2191 (48.1%)	4650 (58.1%)	6841 (54.5%)	
Missing	1600	2546	4146	

[1]Wilcoxon rank-sum p-value; [2]Chi-Square p-value.

Table S3. Pair-wise comparison across adjacent LBR12 level.

	Overall		Chemo-alone		EGFRi		VEGFi	
Comparisons	Hazard Ratio (95% CI)	P-value	Hazard Ratio (95% CI)	P-value	Hazard Ratio (95% CI)	P-value	Hazard Ratio (95% CI)	P-value
PL vs. SL/PL	1.34 (1.02, 1.75)	0.034	1.21 (0.86, 1.72)	0.274	1.45 (0.67, 3.17)	0.348	1.64 (0.94, 2.85)	0.081
SL/PL vs. RL/PL	1.81 (1.55, 2.10)	<.001	1.67 (1.34, 2.09)	<.001	1.71 (1.17, 2.50)	0.006	2.09 (1.59, 2.75)	<.001
RL/PL vs. SL	1.10 (0.96, 1.25)	0.164	1.07 (0.88, 1.31)	0.492	1.28 (0.92, 1.77)	0.143	1.09 (0.86, 1.37)	0.466
SL vs. RL/SL	1.24 (1.17, 1.31)	<.001	1.15 (1.06, 1.26)	0.002	1.32 (1.14, 1.53)	<.001	1.30 (1.18, 1.43)	<.001
RL/SL vs. RL	1.42 (1.32, 1.53)	<.001	1.45 (1.29, 1.63)	<.001	1.51 (1.29, 1.75)	<.001	1.35 (1.18, 1.56)	<.001

LBR12, lesion-based response criteria at 12 weeks; RL, responding lesion; SL, stable lesion; PL, progressing lesion; EGFRi, epidermal growth factor receptor inhibitor; VEGFi: vascular endothelial growth factor inhibitor. Variables adjusted in models: age, gender, ECOG performance status.

Figure S1. Patient classifications by LBR12 vs. RECIST 1.1

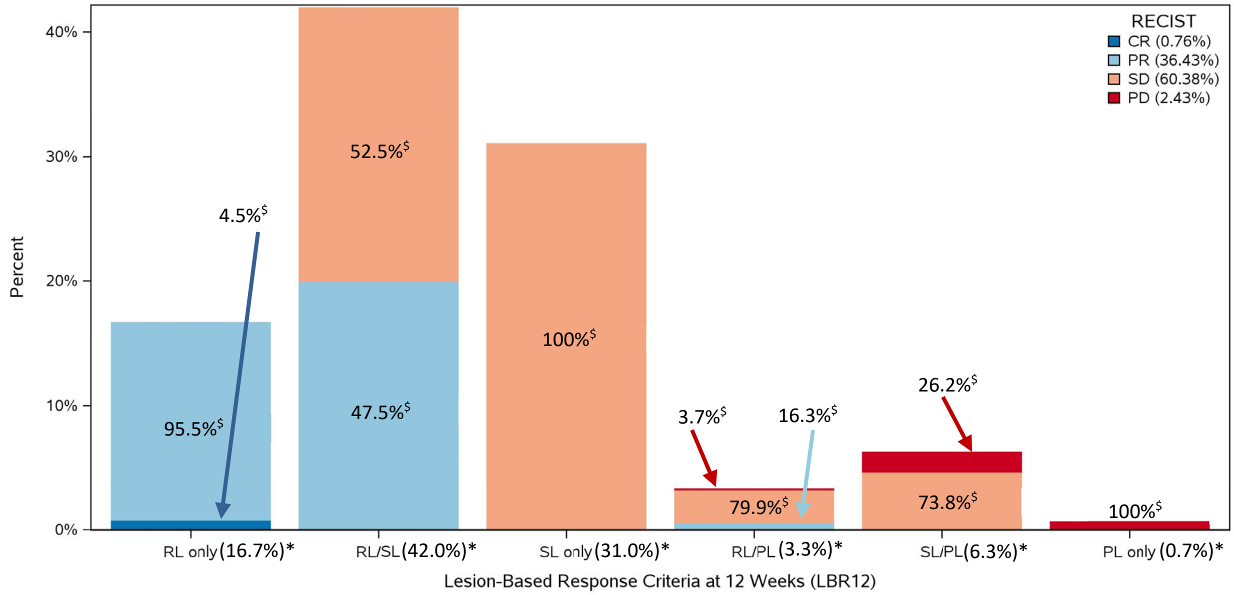

* The percentage indicates the proportion of patients in the specific LBR12 category.

$ The percentage indicates the proportion of patients, within each LBR12 category, with specific RECIST response.

LBR12, lesion-based response criteria at 12 weeks; RECIST 1.1, Response Evaluation Criteria in Solid Tumors version 1.1; RL, responding lesions; SL, stable lesions; PL, progressing lesions; CR, complete response; PR, partial response; SD, stable disease; PD, progression disease.
